# Balanced Crystalloids Versus Normal Saline for Fluid Resuscitation in Pediatric Septic Shock: An Updated Systematic Review and Meta-Analysis

**DOI:** 10.14740/jocmr6685

**Published:** 2026-10-23

**Authors:** Hussein Saeed Alshahrani, Orjuwan Saeed Alruzquwi, Najwa Abdumalik Amiri, Khalid Nawaf Almutairi, Meshari Abdulkarim Almutairi, Leena Turki AldakhilAllah, Shaima Abdulatif Jaber Alsalmi, Fatima Mahmoud Alkandari, Majed Sultan Alenezi, Amal Saeed M. Alrayzah, Mohammad Yaseen Jaha, Sara Ghadeer M. Alghadeer, Abdulaziz Baki Aljohani, Abeer Bassam Alkehaimi, Zainab Ahmed Alsoliabi

**Affiliations:** a Armed Forces Hospital Southern Region, Khamis Mushait, Saudi Arabia; b Ibn Sina National College, Jeddah, Saudi Arabia; c College of Medicine, Fakeeh College for Medical Sciences, Jeddah, Saudi Arabia; d College of Medicine, Majmaah University, Majmaah, Saudi Arabia; e College of Medicine, King Saud bin Abdulaziz University for Health Sciences, Riyadh, Saudi Arabia; f Department of Pediatrics, King Salman Specialist Hospital, Taif, Saudi Arabia; g Faculty of Medicine, Cairo University, Kasr Al Ainy Hospital, Cairo, Egypt; h Department of Pharmacy, Eastern Province Maternity and Children Hospital, Dammam, Saudi Arabia; i College of Medicine, King Khalid University, Abha, Saudi Arabia; j College of Medicine, Imam Abdulrahman Bin Faisal University, Dammam, Saudi Arabia; k College of Nursing, Taibah University, Madinah, Saudi Arabia; l College of Pharmacy, Princess Nourah Bint Abdulrahman University, Riyadh, Saudi Arabia; m Department of Pharmacy, Qatif Central Hospital, Ministry of Health, Dammam, Saudi Arabia

**Keywords:** Balanced crystalloids, Normal saline, Pediatric sepsis, Septic shock, Acute kidney injury, Hyperchloremia, Meta-analysis

## Abstract

**Background:**

Crystalloid fluid is central to pediatric septic shock resuscitation, but the optimal composition is uncertain. Normal saline delivers a supraphysiologic chloride load that may promote hyperchloremic acidosis and renal injury, whereas balanced crystalloids approximate extracellular fluid. Pediatric evidence is mixed, and prior syntheses preceded the large PRoMPT BOLUS trial. We compared balanced crystalloids with normal saline for resuscitation in children with sepsis or septic shock.

**Methods:**

We searched PubMed/MEDLINE, Scopus, Web of Science, and Cochrane Central Register of Controlled Trials (CENTRAL) from inception to April 2026 for randomized controlled trials (RCTs) and observational cohorts comparing balanced crystalloids with 0.9% saline in children (≤ 18 years) with sepsis or septic shock requiring fluid resuscitation. Co-primary outcomes were all-cause mortality and acute kidney injury (AKI). Data were pooled using random-effects models (risk ratios (RR), mean differences (MD); 95% confidence interval (CI)), with trial sequential analysis (TSA) for primary outcomes. The review followed the Preferred Reporting Items for Systematic Reviews and Meta-Analyses (PRISMA) 2020 and was registered in PROSPERO.

**Results:**

Nine studies (five RCTs, four observational cohorts) were included. Balanced crystalloids did not reduce mortality (RR, 0.94; 95% CI, 0.83–1.06; I^2^ = 0%) or AKI (RR, 0.84; 95% CI, 0.61–1.16; I^2^ = 73.5%); TSA showed the required information size was unmet, leaving both inconclusive. Balanced crystalloids significantly reduced renal replacement therapy (RR 0.74; 95% CI, 0.56–0.96; I^2^ = 23.5%) and hyperchloremia (RR, 0.65; 95% CI, 0.61–0.69; I^2^ = 0%). No differences emerged for major adverse kidney events within 30 days (MAKE30) (RR, 1.11; 95% CI, 0.87–1.41), mechanical ventilation (RR, 0.97, 95% CI, 0.85–1.11), or hospital (MD, 1.49 days) and pediatric intensive care unit (PICU) (MD, 0.84 days) length of stay.

**Conclusions:**

In pediatric sepsis or septic shock, balanced crystalloids consistently reduce hyperchloremia and the need for renal replacement therapy but do not significantly reduce mortality, AKI, MAKE30, mechanical ventilation, or length of stay. Incorporating recent pragmatic randomized data supports cautious interpretation, and TSA confirms that the primary-outcome evidence remains inconclusive. Adequately powered, phenotype-informed trials are needed.

## Introduction

Pediatric septic shock is a time-sensitive syndrome in which circulatory failure, cellular metabolic dysfunction, and inflammatory organ injury evolve rapidly after infection. Despite advances in early recognition and intensive care, sepsis remains a major contributor to childhood mortality and critical illness worldwide, with the highest burden in young children and neonates, and with substantial geographic variation in outcomes [1, 2]. Current pediatric sepsis management emphasizes prompt antimicrobial therapy, source control, vasoactive support when indicated, and careful restoration of intravascular volume to reverse hypoperfusion [3]. Within this initial resuscitation window, crystalloid fluid is one of the most frequently delivered interventions, yet the optimal crystalloid composition remains debated.

Normal saline has historically been the first-line resuscitation fluid approach in many emergency departments and pediatric intensive care units because it is inexpensive, widely available, isotonic, and compatible with routine clinical practice. However, 0.9% sodium chloride contains 154 mmol/L of both sodium and chloride, a chloride concentration higher than that of plasma. This nonphysiological chloride load may contribute to hyperchloremic metabolic acidosis, reduced strong ion difference, renal vasoconstriction, impaired renal cortical perfusion, and inflammatory or endothelial effects, particularly when repeated boluses are administered during shock [4, 5]. These concerns are clinically relevant in pediatric septic shock, where acute kidney injury (AKI), fluid imbalance, electrolyte disturbance, and acid–base derangement may compound infection-related organ dysfunction [6, 7].

Balanced crystalloids, including lactated Ringer’s, Plasma-Lyte, Ringer’s acetate, Hartmann’s solution, and Sterofundin, contain lower chloride concentrations and buffer anions such as lactate, acetate, or gluconate. Their composition more closely approximates extracellular fluid and may therefore reduce chloride exposure while preserving acid–base balance [4]. Adult critical-care studies have stimulated interest in balanced fluids, but pediatric extrapolation is imperfect, as children differ in cardiovascular reserve, renal physiology, disease patterns, weight-adjusted fluid exposure, and thresholds for organ support [8]. Pediatric-specific evidence is therefore essential before firm clinical recommendations can be made.

Available pediatric studies have produced mixed findings. Earlier observational cohorts suggested possible renal or survival advantages with balanced crystalloids, although residual confounding by illness severity, site practice, and fluid-selection bias remained difficult to exclude [9, 10]. More recent pediatric randomized controlled trials (RCTs) showed clearer biochemical advantages, particularly lower hyperchloremia and metabolic acidosis, and some trials reported reductions in AKI or renal replacement therapy (RRT); however, most were modest in size and were not primarily powered for mortality [11, 12]. Accordingly, the 2020 Surviving Sepsis Campaign pediatric guidelines issued only a weak recommendation, based on very low-quality evidence, favoring balanced or buffered crystalloids over saline for initial fluid bolus therapy in children with septic shock or sepsis-associated organ dysfunction [3].

Several systematic reviews have attempted to address this gap; however, their conclusions differ according to the included populations, study designs, and search dates [13, 14]. These prior studies preceded the large PRoMPT BOLUS trial, which substantially increased the randomized evidence base and found no significant difference in death, new RRT, or persistent kidney dysfunction between balanced fluids and saline [15]. Therefore, an updated systematic review and meta-analysis is warranted to integrate contemporary randomized and observational evidence. The present review evaluates balanced crystalloids versus normal saline for pediatric sepsis or septic shock requiring fluid resuscitation.

## Materials and Methods

### Protocol and registration

This systematic review and meta-analysis was conducted and reported in accordance with the Preferred Reporting Items for Systematic Reviews and Meta-Analyses (PRISMA) 2020 statement and the Cochrane Handbook for Systematic Reviews of Interventions [16, 17]. The protocol was registered prospectively in the International Prospective Register of Systematic Reviews (PROSPERO; registration identifier CRD420261448477).

### Data sources and search strategy

We systematically searched PubMed/MEDLINE, Scopus, Web of Science, and the Cochrane Central Register of Controlled Trials (CENTRAL) from database inception to April 2026. The search combined controlled vocabulary and free-text terms for the population, intervention, and comparator, structured around the core string: (septic shock OR sepsis) AND (children OR pediatric) AND (balanced crystalloids OR Plasma-Lyte OR lactated ringer) AND (normal saline). To maximize sensitivity, the reference lists of all included studies and of relevant systematic reviews were hand-searched for additional eligible records. The complete database-specific search strategy is provided here (Supplementary Material 1, jocmr.elmerjournals.com).

### Eligibility criteria

Studies were selected according to the following population, intervention, comparator, and outcomes (PICO) framework. Population: children (≤ 18 years) with sepsis or septic shock requiring fluid resuscitation. Intervention: balanced crystalloids (e.g., lactated Ringer’s, Ringer’s acetate, Plasma-Lyte/Plasma-Lyte A, Hartmann’s, or Sterofundin). Comparator: normal saline (0.9% sodium chloride). Outcomes: at least one of all-cause mortality, AKI, metabolic or electrolyte outcomes (including hyperchloremia and acid–base parameters), need for RRT or mechanical ventilation, hospital or pediatric intensive care unit (PICU) length of stay, or adverse events. Both RCTs and observational (cohort) studies were eligible. We excluded adult-only studies; case reports, editorials, and narrative reviews; animal or *in vitro* studies; and studies with insufficient or non-extractable outcome data.

### Study selection

All records were imported into Rayyan, and duplicates were removed. Two reviewers independently screened titles and abstracts and then assessed the full texts of potentially eligible reports against the predefined criteria; disagreements at either stage were resolved by a third reviewer.

### Data extraction

Two reviewers independently extracted data into a standardized form, with discrepancies resolved by consensus. Extracted items comprised study characteristics (first author, year, country, design, setting, sample size, and follow-up), patient characteristics (age, sex, illness severity, focus of infection, and baseline severity or lactate values), the type of balanced fluid and comparator, and all reported outcomes. The primary outcomes were mortality and AKI. Secondary outcomes were major adverse kidney events within 30 days (MAKE30), need for RRT, need for mechanical ventilation, hyperchloremia, and hospital and PICU length of stay. Prespecified acid–base indices (pH, bicarbonate, lactate) and vasopressor requirement were extracted but were reported at heterogeneous time points and in incompatible formats and could not be quantitatively pooled. Where outcomes were reported as medians with interquartile ranges, they were converted to means and standard deviations using established methods [18].

### Quality assessment and certainty of evidence

Two reviewers independently appraised methodological quality, with disagreements resolved by discussion. RCTs were assessed with the Cochrane Risk of Bias 2 (RoB-2) tool across five domains: bias arising from the randomization process, deviations from intended interventions, missing outcome data, measurement of the outcome, and selection of the reported result; results were visualized as traffic-light and weighted summary plots [19]. Observational studies were assessed with the Newcastle–Ottawa Scale (NOS) across the selection, comparability, and outcome domains, with total scores of 7–9 classified as good, 4–6 as fair, and 0–3 as poor quality [20]. In addition, we used the Grading of Recommendations Assessment, Development, and Evaluation (GRADE) criteria to evaluate the quality of the evidence [21, 22].

### Statistical analysis

Meta-analyses were performed using R (version 4.5.0) and RStudio (version 2024.12.1 + 563). Dichotomous outcomes were pooled as risk ratios (RR) and continuous outcomes as mean differences (MD), each with 95% confidence intervals (CI), using the DerSimonian–Laird random-effects model with inverse-variance weighting. Statistical heterogeneity was quantified with the I^2^ statistic and Cochran’s Q test, with I^2^ > 50% regarded as substantial. Prespecified subgroup analyses were performed, where sufficient data were available, according to study design (RCT vs observational) and, for mortality, the timing of outcome assessment (in-hospital, 30-day, and 90-day); differences between subgroups were examined with the χ^2^ test. The robustness of the primary outcomes was tested in leave-one-out sensitivity analyses. Because fewer than 10 studies contributed to every outcome, funnel plots and Egger’s test were not applied; small-study effects were explored with a Doi plot and the Luis Furuya-Kanamori (LFK) index, interpreting LFK ≤ 1 as no asymmetry, > 1 to 2 as minor asymmetry, and > 2 as major asymmetry [23].

### Trial sequential analysis (TSA)

Because cumulative meta-analysis is prone to type I error from repeated significance testing and to type II error when the accrued sample is small, TSA was performed for the two primary outcomes, all-cause mortality and AKI, using the TSA software (Copenhagen Trial Unit). The required information size (RIS) was calculated assuming a two-sided α of 5% and a power of 80% (β = 20%), based on the control-group event proportion and an *a priori* plausible relative risk reduction, with adjustment for heterogeneity (diversity). O’Brien–Fleming monitoring boundaries and the cumulative Z-curve were constructed; conclusive evidence was inferred when the Z-curve crossed a monitoring or futility boundary or when the cumulative information exceeded the RIS, and the evidence was otherwise considered inconclusive. TSA was conducted for all studies combined and, separately, for the RCTs alone. All analyses were performed using TSA version 0.9 beta.

### Institutional Review Board approval

Institutional Review Board approval was not applicable because this study synthesized previously published aggregate data and did not involve direct human or animal participant research or identifiable individual-level data.

### Ethical compliance with human/animal study

This systematic review did not involve direct human or animal experimentation. It was conducted in accordance with applicable research-integrity and publication-ethics standards.

## Results

### Literature search

The electronic search of PubMed/MEDLINE, Scopus, Web of Science, and Cochrane CENTRAL (database inception to April 2026), supplemented by screening of reference lists and relevant systematic reviews, retrieved 270 records. After removal of 76 duplicates, 194 titles and abstracts were screened, and 30 full-text articles were assessed for eligibility. Nine studies fulfilled the eligibility criteria and were included in the qualitative synthesis and meta-analysis. Reasons for full-text exclusion are detailed in the PRISMA 2020 flow diagram (Fig. 1).

**Figure 1:**
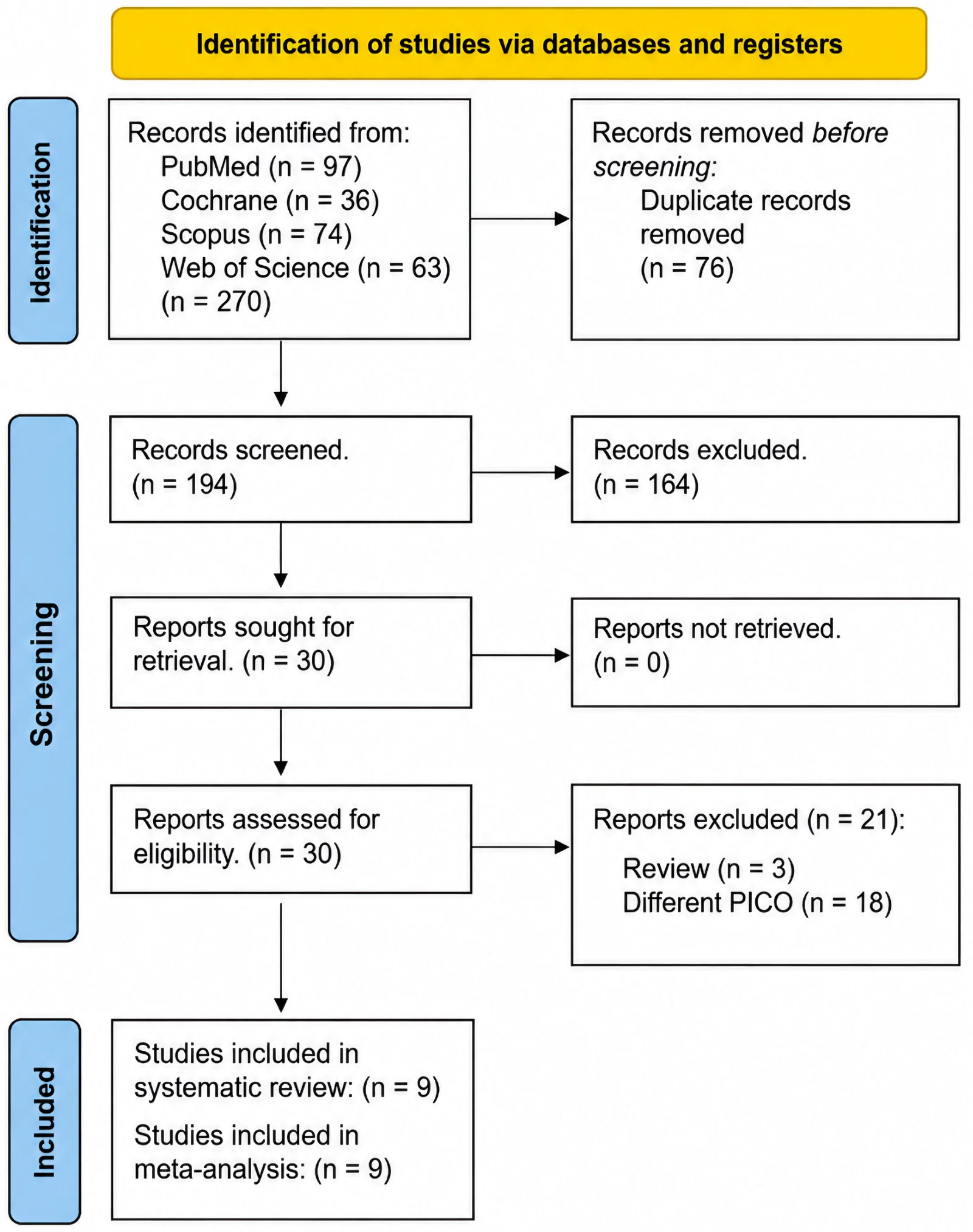
PRISMA 2020 flow diagram of the study-selection process. PICO: population, intervention, comparator, and outcomes; PRISMA: Preferred Reporting Items for Systematic Reviews and Meta-Analyses.

### Characteristics of included studies

The nine included studies comprised five RCTs and four observational cohort studies published between 2017 and 2026 (Table 1) [9–11, 15, 24–28]. They were conducted in Thailand, India, the USA, Colombia, and a multinational network (USA, Canada, Australia, New Zealand, and Costa Rica), and together enrolled children aged 1 month to 18 years with sepsis or septic shock requiring fluid resuscitation. Analytic cohorts ranged from 22 patients (Trepatchayakorn et al 2021 [26]) to more than 10,000 (Emrath et al 2017 [9]) and were dominated by two large, pragmatic trials (Balamuth et al, 2026 [15] (n = 8,482), and Sankar et al 2023 [11] (n = 708)). Balanced-crystalloid arms received lactated Ringer’s, Ringer’s acetate, Plasma-Lyte/Plasma-Lyte A, Hartmann’s, or Sterofundin solutions, compared with 0.9% sodium chloride. Reported outcomes included mortality, AKI, major adverse kidney events (MAKE30), RRT, mechanical ventilation, hyperchloremia, and hospital/PICU length of stay; baseline demographic and illness-severity characteristics were broadly comparable between groups (Table 2). Prespecified acid–base indices (pH, bicarbonate, lactate) and vasopressor requirement were reported at heterogeneous time points and in incompatible formats and could not be quantitatively pooled.

**Table 1:** Summary of Included Studies

Study	Study design	Country	Time frame	Follow-up (days)	Sample size	Type of intervention	Inclusion criteria	Primary outcomes
Samransamruajkit et al, 2017 [27]	Prospective observational study	Thailand	June 2013 to March 2014	NA	35 (20 NSS, 15 RLS)	LR	Children 1 month to 15 years with severe sepsis or septic shock	Effectiveness of balanced salt vs. 0.9% NSS on morbidity and mortality
Anantasit et al, 2020 [28]	Double-blind RCT	Thailand	January 2015 to December 2017	NA	61 (31 NS, 30 BS)	Ringer’s acetate	Children 1 month to 18 years with septic shock needing fluid resuscitation	Incidence of hyperchloremic metabolic acidosis within 48 h
Balamuth et al, 2019 [24]	Open-label pragmatic RCT	USA	January to August 2018	90	50 (26 NS, 24 LR)	LR	Children > 6 months to < 18 years with suspected septic shock	Feasibility (enrollment proportion, EFIC acceptability, and adherence)
Balamuth et al, 2026 [15]	Pragmatic multicenter clinical trial (RCT)	USA, Canada, Australia, NZ, Costa Rica	August 25, 2020 to October 31, 2025	90	8,482 analyzed (4,247 NS, 4,235 balanced)	LR, PL, or Hartmann’s solution	Children 2 months to < 18 years with suspected septic shock and abnormal perfusion	Major adverse kidney event (MAKE30) at 30 days or hospital discharge
Emrath et al, 2017 [9]	Observational matched cohort review	USA	January 2004 to December 2012	3	36,908 (PICU admissions)	Balanced fluids	Children 0–18 years with pediatric severe sepsis or septic shock in a PICU	In-hospital mortality
Sankar et al, 2023 [11]	Parallel-group, blinded multicenter trial (RCT)	India	April 10, 2017 to Jan 31, 2020	28	708 (357 saline, 351 MES)	PL A	Children up to 15 years of age with septic shock	New and/or progressive acute kidney injury within first 7 days
Fernández-Sarmiento 2021 [25]	Observational analytical cohort (propensity matched)	Colombia	April 1, 2018 to April 30, 2019	NA	99 (35 unbalanced, 64 balanced)	LR; PL	Children 1 month to 17 years with sepsis-associated organ dysfunction or shock	Presence of acute kidney injury
Trepatchayakorn et al, 2021 [26]	RCT	Thailand	November 2016 to November 2019	NA	42	Ringer lactate; Sterofundin	Pediatric sepsis patients requiring fluid bolus	Comparison of acid–base status after fluid bolus therapy
Weiss et al, 2017 [10]	Matched retrospective cohort study	USA	2000 to 2013	30	12,529 total (4,234 matched)	LR	Children < 18 years with severe sepsis/septic shock receiving fluid boluses	All-cause 30-day hospital mortality

BS: balanced salt solution; EFIC: exception from informed consent; LR: lactated Ringer; MAKE30: major adverse kidney event within 30 days; MES: multiple electrolyte solution; NZ: New Zealand; NS: normal saline; NSS: normal saline solution; NA: not available; PICU: pediatric intensive care unit; PL: Plasma-Lyte; RCT: randomized controlled trial; RLS: Ringer lactate solution.

**Table 2:** Baseline Patient Characteristics in Each Study

Study ID	Groups	Patient demographics			Hemodynamic parameters, mean ± SD			Focus of infection, n (%)		
		Age (years)	Weight (kg)	Male, n (%)	SOFA score	PRISM III score	Lactate (mmol/L)	Pulmonary	Urinary	Skin/soft tissue
Samransamruajkit et al, 2017 [27]	BS	6.4 ± 4.8	24.28 ± 17.7	9 (60)	6.67 ± 2.02	5.40 ± 4.64	NA	18 (51.4)	4 (11.4)	5 (14.3)
	NS	4.2 ± 4.9	17.27 ± 14.89	12 (60)	8.30 ± 2.64	8.75 ± 4.64	NA			
Anantasit et al, 2020 [28]	BS	6.03 ± 8.33	NA	14 (46.7)	NA	7.83 ± 7.79	1.83 ± 1.09	8 (26.7)	NA	NA
	NS	6.25 ± 7.31	NA	22 (70.9)	NA	9.67 ± 10.89	2.4 ± 1.4	5 (16.1)	NA	NA
Balamuth et al, 2019 [24]	BS	5.4 ± 6.62	NA	13 (54)	NA	NA	NA	5 (21)	4 (17)	2 (8)
	NS	9.53 ± 10.8	NA	14 (54)	NA	NA	NA	5 (19)	3 (12)	3 (12)
Balamuth et al, 2026 [15]	BS	7.47 ± 7.42	NA	2166 (51.2)	NA	NA	2.07 ± 1.26	46.6%	NA	NA
	NS	7.47 ± 7.42	NA	2136 (50.4)	NA	NA	2.07 ± 1.26		NA	NA
Emrath et al, 2017 [9]	BS	6.0 ± 6.1	NA	1308 (54.5)	NA	NA	NA	NA	NA	NA
	NS	7.4 ± 6.3	NA	15891 (52.7)	NA	NA	NA	NA	NA	NA
Sankar et al, 2023 [11]	BS	4.67 ± 5.96	NA	201 (57)	7 ± 4.47	NA	NA	149 (42)	7 (2)	5 (1)
	NS	5.17 ± 5.59	NA	205 (58)	7 ± 4.47	NA	NA	129 (36)	10 (3)	6 (2)
Fernandez-Sarmiento 2021 [25]	BS	NA^a^	NA	35 (55.5)	NA	NA^a^	NA	37 (58.73)	3 (4.76)	3 (4.76)
	NS		NA	19 (55.5)	NA		NA	19 (52.78)	4 (11.11)	4 (11.11)
Trepatchayakorn et al, 2021 [26]	BS	NA	NA	24 (57.1)	NA	4	4.1 ± 5.57	NA	NA	NA
	NS	NA	NA		NA		2.07 ± 1.43	NA	NA	NA
Weiss et al, 2017 [10]	BS	8 ± 10.39	NA	1,119 (53)	NA	NA	NA	NA	NA	NA
	NS	7.33 ± 9.65	NA	1,149 (54)	NA	NA	NA	NA	NA	NA

^a^For this study (Fernandez-Sarmiento 2021 [25]), the published overall-cohort age was median 9.9 months (IQR 4.9–22.2); arm-specific age and PRISM III summary values could not be independently verified and are shown as NA. Other mean ± SD entries are reproduced as shown in the source table. BS: balanced salt solution; NS: normal saline; NA: not available; SOFA: sequential organ failure assessment; SD: standard deviation.

### Risk of bias assessment

Among the five RCTs, risk of bias appraised with the Cochrane RoB-2 tool was low overall for every trial (Supplementary Material 2, jocmr.elmerjournals.com). Some concerns were limited to measurement of the outcome in the two open-label trials (Balamuth et al 2019 [24] and Balamuth et al 2026 [15]); all remaining domains were rated low risk. The four observational studies, assessed with the NOS, were of good quality (Emrath et al 2017 [9], Weiss et al 2017 [10], Samransamruajkit et al 2017 [27]) or fair quality (Fernandez-Sarmiento et al 2021 [25]) (Supplementary Material 3, jocmr.elmerjournals.com).

### Primary outcomes

#### All-cause mortality

Nine studies reported mortality. Balanced crystalloids were not associated with a significant reduction in mortality compared with normal saline (RR 0.94; 95% CI, 0.83–1.06; P = 0.29), with no heterogeneity (I^2^ = 0%) (Fig. 2). The estimate was consistent across study designs (RCTs: RR 1.02; 95% CI, 0.86–1.20; observational: RR 0.88; 95% CI, 0.77–1.00; subgroup difference P = 0.17) (Supplementary Material 4, jocmr.elmerjournals.com) and across assessment time points (in-hospital RR 0.91; 95% CI, 0.82–1.02; 30-day RR 1.08; 95% CI, 0.77–1.53; 90-day RR 1.09; 95% CI, 0.82–1.44; subgroup difference P = 0.37) (Supplementary Material 5, jocmr.elmerjournals.com). The pooled result was robust in leave-one-out analysis (RR range 0.91–1.02) (Supplementary Material 6, jocmr.elmerjournals.com). The Doi plot for all-cause mortality looks symmetrical, with an LFK index of 0.03, well within ±1. This shows no asymmetry, so small-study effects or publication bias are unlikely. (Supplementary Material 7, jocmr.elmerjournals.com)

**Figure 2:**
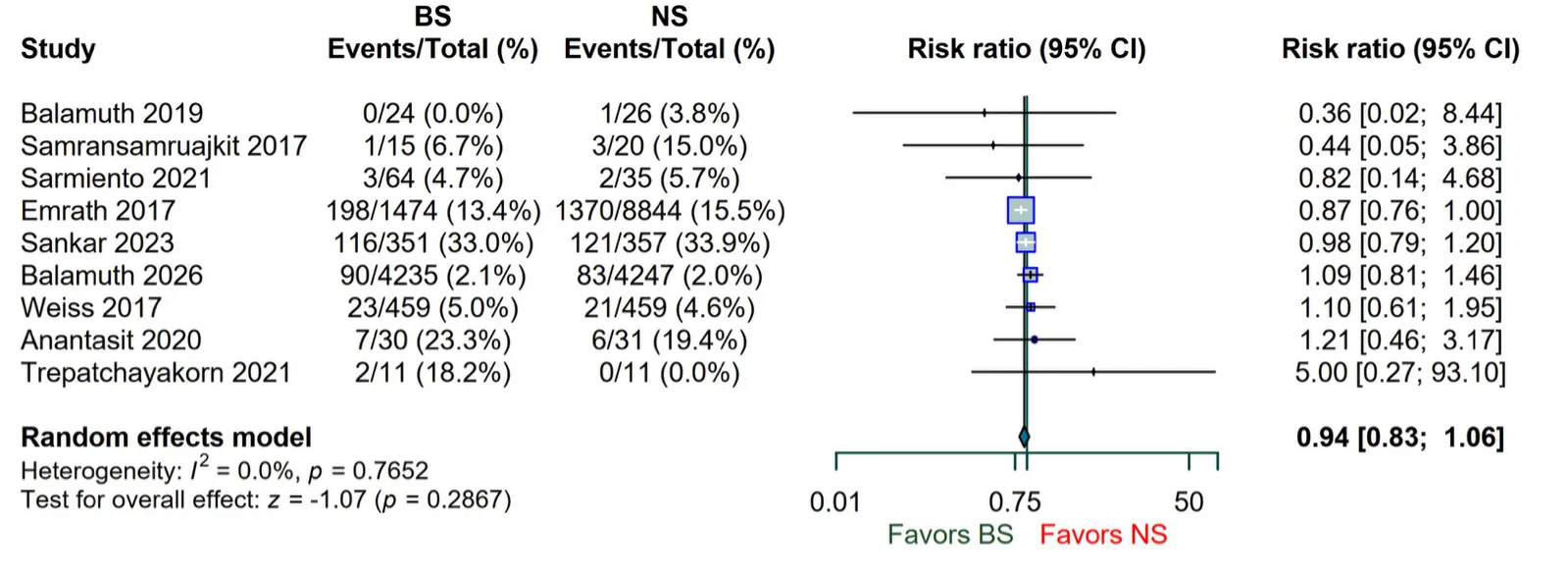
Forest plot of all-cause mortality. BS: balanced salt solution; NS: normal saline; CI: confidence interval.

TSA (Fig. 3a) showed that the cumulative Z-curve did not cross the conventional (Z = ±1.96) or the trial-sequential monitoring boundaries, and the accrued information did not reach RIS (RIS = 41,533). The analysis restricted to RCTs was likewise inconclusive (RIS = 33,821) (Fig. 3b). The current evidence is therefore insufficient to confirm or exclude a clinically important mortality difference, and a true effect cannot be ruled out with the available sample.

**Figure 3:**
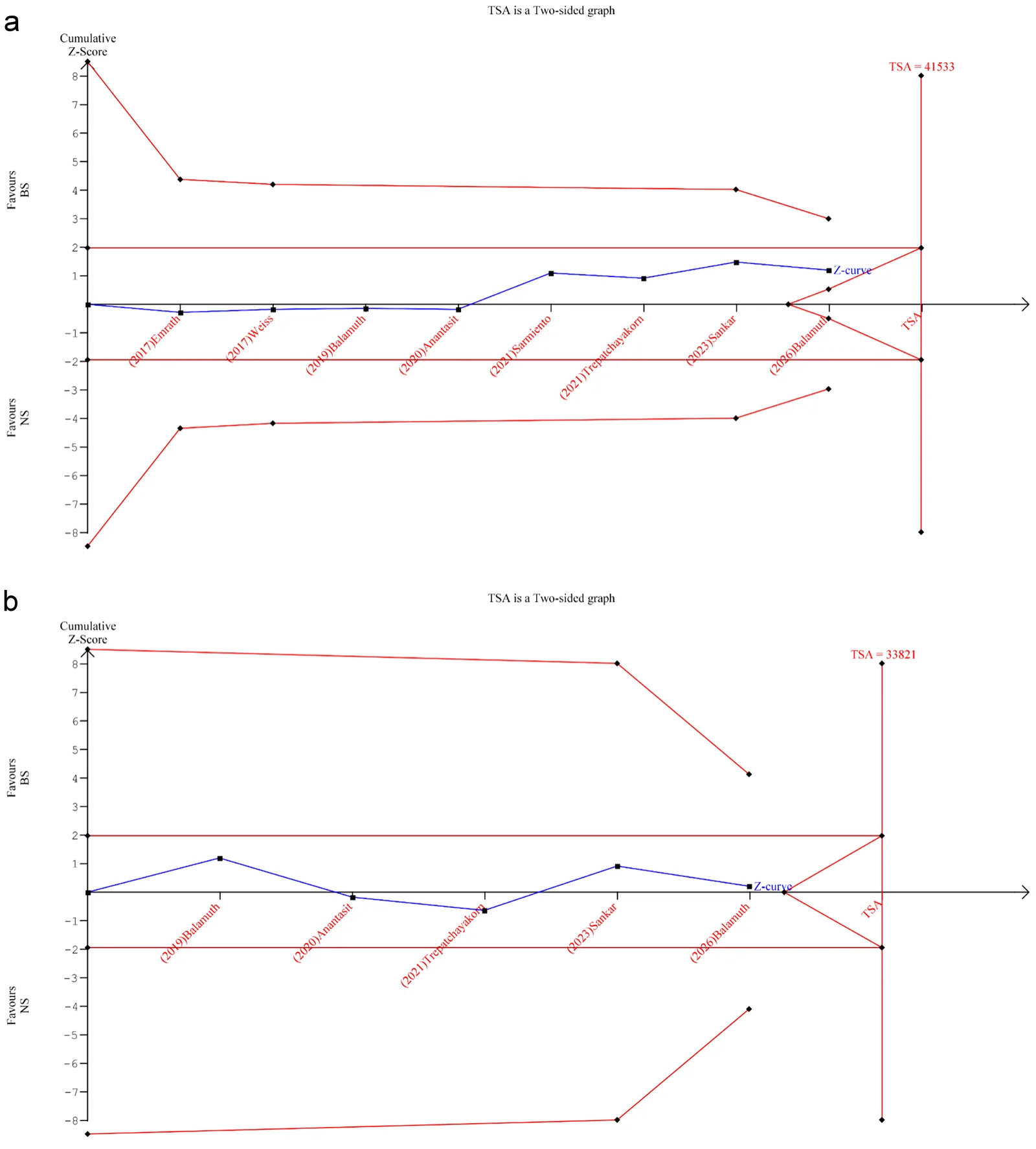
Trial sequential analysis of all-cause mortality. (a) Analysis including all eligible studies. (b) Analysis restricted to randomized controlled trials. BS: balanced salt solution; NS: normal saline; TSA: trial sequential analysis.

#### AKI

Eight studies contributed to the AKI analysis. The pooled estimate favored balanced crystalloids but did not reach statistical significance (RR 0.84; 95% CI, 0.61–1.16; P = 0.30), with substantial heterogeneity (I^2^ = 73.5%) (Fig. 4). Subgrouping by design did not explain the heterogeneity (RCTs: RR 0.94; 95% CI, 0.58–1.53; I^2^ = 69.1%; observational: RR 0.74; 95% CI, 0.42–1.32; I^2^ = 82.5%; subgroup difference P = 0.54) (Supplementary Material 8, jocmr.elmerjournals.com). Both the null effect and the high heterogeneity persisted across all leave-one-out iterations (RR 0.78–0.94; I^2^ = 59–77%) (Supplementary Material 9, jocmr.elmerjournals.com), indicating that no single study accounted for the inconsistency. Doi-plot assessment showed only minor asymmetry (LFK index −1.26), making major publication bias unlikely (Supplementary Material 10, jocmr.elmerjournals.com).

**Figure 4:**
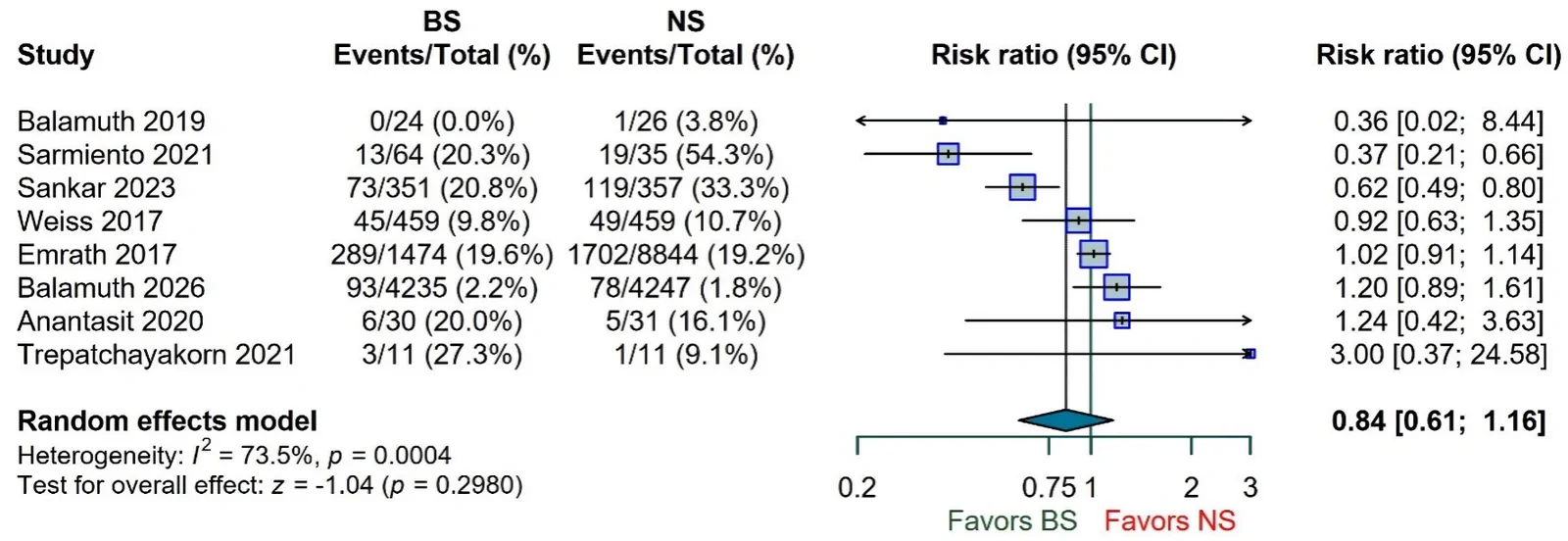
Forest plot of acute kidney injury. BS: balanced salt solution; NS: normal saline; CI: confidence interval.

TSA (Fig. 5a) demonstrated that the Z-curve remained within the monitoring boundaries and that the accrued sample was far below the RIS (RIS = 41,744 for all studies and 34,376 for RCTs only) (Fig. 5b). The evidence for AKI is therefore inconclusive, reflecting both clinical heterogeneity and insufficient information size.

**Figure 5:**
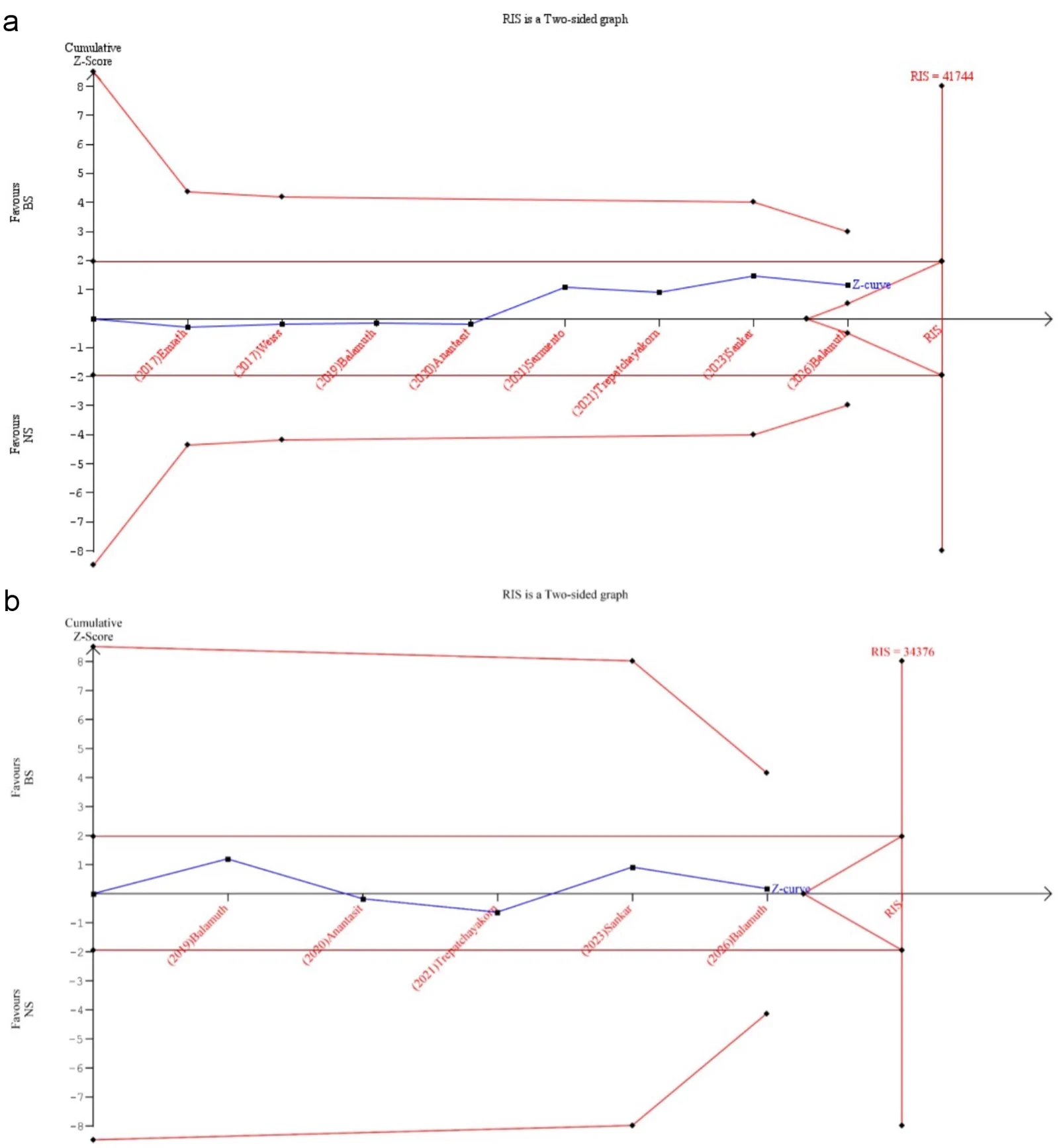
Trial sequential analysis of acute kidney injury. (a) Analysis including all eligible studies. (b) Analysis restricted to randomized controlled trials. BS: balanced salt solution; NS: normal saline; RIS: required information size.

### Secondary outcomes

#### Major adverse kidney events (MAKE30)

Two RCTs (Balamuth et al 2019 [24] and Balamuth et al 2026 [15]; 8,532 children) reported MAKE30. There was no significant difference between balanced crystalloids and normal saline (RR 1.11; 95% CI, 0.87–1.41; P = 0.40; I^2^ = 0%) (Supplementary Material 11, jocmr.elmerjournals.com), with the pooled estimate driven by the large Balamuth et al 2026 [16] trial.

#### Need for RRT

Eight studies (four RCTs and four observational cohorts) reported RRT. Overall, balanced crystalloids reduced RRT (RR 0.74; 95% CI, 0.56–0.96; P = 0.03; I^2^ = 23.5%). The RCT subgroup showed a significant reduction (RR 0.67; 95% CI, 0.47–0.97; I^2^ = 0%; P = 0.033), whereas the observational subgroup did not (RR 0.63; 95% CI, 0.30–1.33; I^2^ = 39.4%; P = 0.228); there was no evidence of a subgroup difference (P = 0.8815) (Supplementary Material 12, jocmr.elmerjournals.com).

#### Need for mechanical ventilation

Three studies reported the need for mechanical ventilation, with no difference between groups (RR 0.97; 95% CI, 0.85–1.11; P = 0.65; I^2^ = 0%) (Supplementary Material 13, jocmr.elmerjournals.com).

#### Hyperchloremia

Four RCTs (Anantasit et al 2020 [28], Balamuth et al 2019 [24], Balamuth et al 2026 [25], and Sankar et al 2023 [11]) reported the occurrence of hyperchloremia. Balanced crystalloids substantially and significantly reduced its incidence (RR 0.65; 95% CI, 0.61–0.69; P < 0.0001) with no heterogeneity (I^2^ = 0%) (Fig. 6).

**Figure 6:**
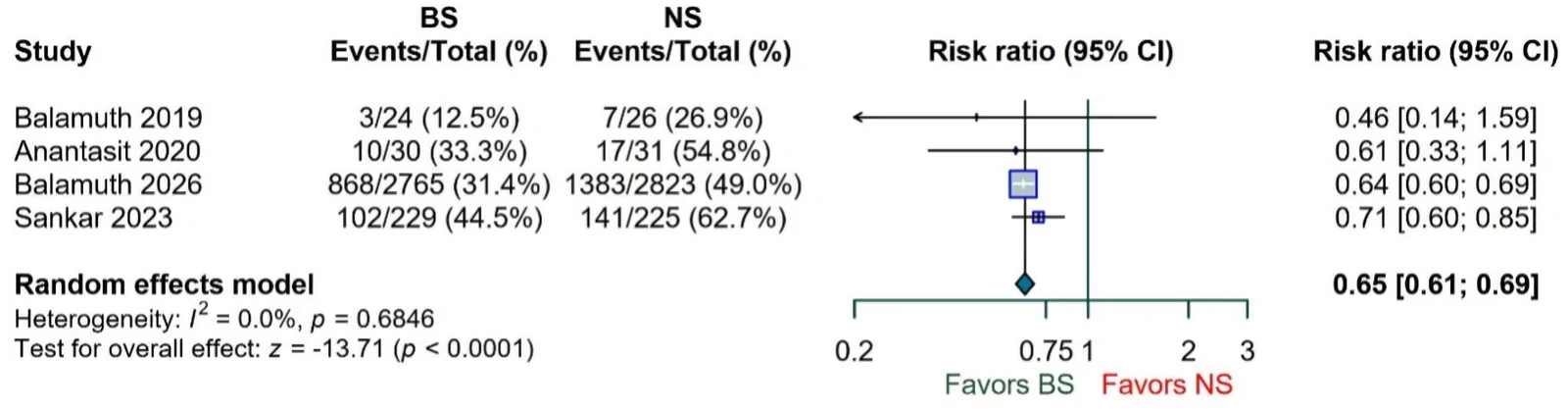
Forest plot of hyperchloremia. BS: balanced salt solution; NS: normal saline; CI: confidence interval.

#### Length of stay

Neither hospital length of stay (four studies; MD 1.49 days; 95% CI, –1.65 to 4.62; P = 0.35; I^2^ = 65.2%) (Supplementary Material 14, jocmr.elmerjournals.com) nor PICU length of stay (three studies; MD 0.84 days; 95% CI, –0.27 to 1.95; P = 0.14; I^2^ = 0%) (Supplementary Material 15, jocmr.elmerjournals.com) differed significantly between balanced crystalloids and normal saline.

### Certainty of evidence (GRADE)

Risk of bias was judged not serious across the evaluated outcomes. Uncertainty in the evidence was driven mainly by imprecision, with additional inconsistency for AKI and hospital length of stay; TSA remained inconclusive for all-cause mortality and AKI. Using GRADE, the certainty of evidence was moderate for all-cause mortality (both all-study and RCT-only analyses), MAKE30, RRT, mechanical ventilation, and PICU length of stay; low for AKI (both all-study and RCT-only analyses) and hospital length of stay; and high for hyperchloremia (Supplementary Material 16, jocmr.elmerjournals.com).

## Discussion

This meta-analysis comprehensively examined balanced crystalloids versus normal saline for pediatric septic shock resuscitation and included nine studies comprising five RCTs and four observational cohort studies. Our study revealed several key findings regarding clinical outcomes. Balanced crystalloids were associated with a nonsignificant reduction in mortality compared with normal saline, with no heterogeneity observed among included studies. Similarly, AKI showed no statistically significant difference, though the pooled estimate favored balanced crystalloids. Nevertheless, balanced crystalloids demonstrated significant benefits in reducing the need for RRT and substantially reduced hyperchloremia incidence with a RR of 0.74 and 0.65, respectively. No significant differences were observed for MAKE30, mechanical ventilation, hospital length of stay, or PICU length of stay.

The absence of a significant mortality reduction in the balanced crystalloids group is essential for interpretation. Initial interest in balanced crystalloids use for pediatric sepsis was largely supported by biological rationale and observational evidence; however, the current updated data indicate that a definitive survival benefit has not been established. This conclusion is consistent with the study by Vijendra et al [13], who found no significant difference in hospital mortality after pooling randomized and observational pediatric studies [13]. Chandelia et al’s RCT-only synthesis also showed no significant in-group difference regarding mortality reduction [14]. The present analysis extends both reviews by adding PRoMPT BOLUS, which contributed 8,482 analyzed children and found no significant difference in its kidney-centered composite or death [15]. Thus, the mortality signal now favors a neutral interpretation supported by contemporary randomized evidence.

A key reason for this change is the weight of newer pragmatic evidence. The PRoMPT BOLUS trial tested a broader real-world strategy using lactated Ringer’s, Plasma-Lyte, or Hartmann’s solution across several countries [15]. They suggested that balanced fluids may modify chloride-related and kidney-support outcomes without reliably improving survival. Similar patterns have been observed in adult critical care. The SPLIT, PLUS, and BaSICS trials did not demonstrate major mortality or AKI advantages from buffered or balanced fluids, whereas adult sepsis subgroup data and individual patient-data meta-analysis suggest that any benefit is probably modest and may vary by subgroup [29–33]. This broader literature suggests that crystalloid composition alone is unlikely to dominate outcomes in complex shock states.

Renal outcomes require particularly careful interpretation. The significant reduction in RRT, combined with a nonsignificant AKI result and neutral MAKE30 result, suggests that balanced crystalloids may reduce progression to more severe kidney failure in some children without consistently preventing all AKI events. Chandelia et al [14] reported lower AKI after sensitivity analysis and lower RRT with balanced salt solutions, while Vijendra et al [13] found no overall AKI difference but lower RRT in the RCT subgroup. Our updated findings reconcile these differences by showing a stronger signal for RRT than for AKI. This distinction is clinically plausible because AKI in septic shock is multifactorial, involving hypoperfusion, inflammation, endothelial dysfunction, microcirculatory changes, nephrotoxic exposure, and fluid accumulation rather than chloride exposure alone [34]. Heterogeneity in AKI definitions, timing of creatinine measurement, baseline renal reserve, and fluid volumes may also explain why the AKI estimate remained inconsistent.

Regarding hyperchloremia findings, the results observed were expected because saline delivers a supraphysiologic chloride concentration, whereas balanced crystalloids contain lower chloride and metabolizable buffer anions [7]. Evidence from pediatric populations beyond septic shock also indicates that balanced solutions may improve acid–base parameters. A previous trial by Lehr et al [35] found improved pH and bicarbonate after balanced-fluid bolus therapy in critically ill children. Furthermore, the SPLYT-P trial showed less chloride rise with buffered solutions in the PICU, although clinical outcome evidence remained limited [12]. The present review strengthens this biochemical conclusion by showing a large, consistent reduction in hyperchloremia with no heterogeneity. Nevertheless, the dissociation between reduced hyperchloremia and unchanged mortality or length of stay indicates that correction of a laboratory abnormality is not necessarily sufficient to change downstream outcomes. Hyperchloremia may be a mediator in selected high-risk patients, but in many children, it may also function as a marker of illness severity, total fluid exposure, or treatment context.

Notably, the lack of measurable benefits in mechanical ventilation and length of stay is clinically reasonable. These outcomes are affected by infection source, timing of antibiotics, vasoactive support, respiratory comorbidity, sedation, local admission practices, and discharge readiness. Fluid type is only one element of hemodynamic resuscitation. The updated Cochrane review of buffered solutions in critically ill adults and children similarly found no clear overall mortality advantage, while BEST-Living suggested that the effect of balanced crystalloids may be modest and context dependent [31, 36]. Therefore, the present pediatric findings should not be interpreted as evidence that fluid choice is irrelevant; rather, they show that the measurable benefits of balanced crystalloids are concentrated in chloride exposure and possibly severe renal support, not in broad short-term clinical endpoints.

### Strengths and limitations

Several strengths increase the value of this updated synthesis. It incorporates the most recent pediatric evidence, including PRoMPT BOLUS, and therefore provides a more current estimate than earlier reviews. It also separates randomized and observational evidence, evaluates the timing of mortality assessment, uses leave-one-out analyses, and includes TSA for the two primary outcomes. These methods are important because conventional meta-analysis may overestimate certainty when repeated testing is performed before the accrued sample reaches the RIS [37]. The inclusion of TSA is therefore a major interpretive strength, as it indicates that the mortality and AKI evidence remains statistically inconclusive despite the larger sample. This prevents overinterpretation of neutral findings as proof of equivalence and highlights that imprecision persists even after the incorporation of large, pragmatic data.

Nonetheless, limitations should be acknowledged. First, the included studies differed in design, setting, age range, illness severity, follow-up duration, resuscitation protocols, and balanced-fluid formulation. In addition, Lactated Ringer’s, Ringer’s acetate, Plasma-Lyte, Hartmann’s solution, and Sterofundin were grouped together, although their electrolyte content and buffers are not identical. Moreover, several trials were small or designed for feasibility or biochemical endpoints rather than definitive clinical outcomes. Observational cohorts add external validity but remain vulnerable to residual confounding and treatment-selection bias. AKI definitions were also inconsistent, acid–base indices were reported at incompatible time points, and vasopressor requirements could not be pooled. Hospital length of stay showed substantial heterogeneity, and TSA indicated that further evidence is still required. Finally, publication bias could not be examined reliably for most outcomes as fewer than 10 studies contributed to several pooled analyses.

### Clinical implications and future recommendations

From a clinical perspective, balanced crystalloids can reasonably be favored when the aim is to minimize chloride load and reduce hyperchloremia, particularly in children with evolving metabolic acidosis, high anticipated fluid exposure, or increased renal vulnerability. However, the current evidence does not justify presenting balanced crystalloids as definitively superior for survival or overall AKI prevention. Normal saline remains an acceptable option when balanced fluids are unavailable, costly, or restricted by local logistics, particularly when careful reassessment after each bolus is maintained. This balanced interpretation is compatible with pediatric guideline caution and with adult and mixed critical-care evidence showing either neutral outcomes or modest context-dependent effects [29–32, 36, 38].

Pragmatic pediatric feasibility investigations have already shown that fluid-type randomization can be embedded within emergency sepsis care, supporting the feasibility of larger and more targeted trials [24]. Future studies should focus less on whether one fluid is universally superior and more on which pediatric phenotypes benefit most. Important subgroups include children with baseline hyperchloremia, metabolic acidosis, early AKI, high lactate, malnutrition, severe shock, nephrotoxin exposure, or large cumulative chloride burden. Studies should standardize AKI definitions, separate bolus from maintenance fluids, report total chloride exposure, and include outcomes such as vasopressor-free days, organ-support-free days, fluid balance, renal recovery, and longer-term kidney function. Economic and implementation analyses are also needed, especially in low-resource settings where balanced fluids may be less accessible than saline.

### Conclusions

In children with sepsis or septic shock requiring fluid resuscitation, balanced crystalloids consistently reduce hyperchloremia and may reduce the need for RRT. However, this updated evidence does not demonstrate significant reductions in mortality, overall AKI, MAKE30, mechanical ventilation, or length of stay. The addition of recent large, randomized evidence, particularly PRoMPT BOLUS, supports a more cautious conclusion than some earlier syntheses. Balanced crystalloids are physiologically rational and often clinically preferable for reducing chloride exposure, but their major patient-centered benefits remain unproven. Further adequately powered, phenotype-informed trials are required to define when biochemical advantages translate into meaningful clinical improvement.

## Supplementary Material

Suppl 1 Detailed search strategy for each database.

Suppl 2 Risk-of-bias assessment of the included randomized controlled trials.

Suppl 3 Newcastle–Ottawa Scale assessment of the included observational cohort studies.

Suppl 4 Subgroup analysis of all-cause mortality according to study design.

Suppl 5 Subgroup analysis of all-cause mortality according to mortality assessment time point.

Suppl 6 Leave-one-out sensitivity analysis of all-cause mortality.

Suppl 7 Doi plot of all-cause mortality.

Suppl 8 Subgroup analysis of acute kidney injury according to study design.

Suppl 9 Leave-one-out sensitivity analysis of acute kidney injury.

Suppl 10 Doi plot of acute kidney injury.

Suppl 11 Forest plot of major adverse kidney events within 30 days.

Suppl 12 Subgroup analysis of renal replacement therapy according to study design.

Suppl 13 Forest plot of the need for mechanical ventilation.

Suppl 14 Forest plots of hospital length of stay.

Suppl 15 Forest plots of pediatric intensive care unit length of stay.

Suppl 16 Summary of findings and quality of evidence based on the GRADE system.

## Data Availability

All data generated or analyzed during this study are included in this article and its supplementary information files.

## Abbreviations

AKI: acute kidney injury
CENTRAL: Cochrane Central Register of Controlled Trials
CI: confidence interval
LFK: Luis Furuya-Kanamori
MAKE30: major adverse kidney events within 30 days
MD: mean difference
NOS: Newcastle-Ottawa Scale
PICU: pediatric intensive care unit
PICO: population, intervention, comparator, and outcomes
PRISMA: Preferred Reporting Items for Systematic Reviews and Meta-Analyses
PROSPERO: International Prospective Register of Systematic Reviews
RCT: randomized controlled trial
RIS: required information size
RoB 2: Risk of Bias 2
RR: risk ratio
RRT: renal replacement therapy
TSA: trial sequential analysis
