# Supplementary material for: Balanced Crystalloids Versus Normal Saline for Fluid Resuscitation in Pediatric Septic Shock: An Updated Systematic Review and Meta-Analysis
Source: J Clin Med Res. 2026 Oct 23;18(10):770–83. doi: 10.14740/jocmr6685 (PMC13719638; doi:10.14740/jocmr6685)
Supplement: Supplementary file 1 [file jocmr-18-10-770-s001.docx]

**Suppl 1**. Detailed search strategy for each database.

| Databases | Search strategies | Results | Limitations |
| --- | --- | --- | --- |
| PubMed | (sepsis OR "septic Shock") AND (pediatric OR Children) AND ("multiple electrolytes solution" OR MES OR balanced OR BS OR crystalloid OR buffered OR Plasmalyte OR "Ringer's Lactate" OR LR OR ringer funding OR Hartmann's OR sterio funding OR Kabilyte OR Normosol) AND ("normal saline" OR saline OR "0.9% saline" OR "0.9% sodium chloride" OR "0.9% NaCl") | 97 | No limitations applied |
| Cochrane Library | (sepsis OR "septic Shock") AND (pediatric OR Children) AND ("multiple electrolytes solution" OR MES OR balanced OR BS OR crystalloid OR buffered OR Plasmalyte OR "Ringer's Lactate" OR LR OR ringer funding OR Hartmann's OR sterio funding OR Kabilyte OR Normosol) AND ("normal saline" OR saline OR "0.9% saline" OR "0.9% sodium chloride" OR "0.9% NaCl") | 36 | No limitations applied |
| Scopus | TITLE-ABS-KEY ((sepsis OR "septic shock") AND  (pediatric OR paediatric OR child* OR infant* OR neonat* OR adolescent*) AND ("multiple electrolytes solution" OR "multiple electrolyte solution" OR MES OR balanced OR "balanced crystalloid*" OR "balanced solution*" OR buffered OR "buffered crystalloid*" OR plasmalyte OR "Plasma-Lyte" OR "Plasma Lyte"  OR "Ringer's lactate" OR "lactated Ringer*" OR LR  OR Hartmann* OR "Hartmann's solution"  OR Sterofundin OR Ringerfundin OR Kabilyte OR Normosol) AND ("normal saline" OR saline OR "0.9% saline" OR "0.9% sodium chloride" OR "0.9% NaCl")) | 74 | No limitations applied |
| WOS | TS=(sepsis OR "septic Shock") AND (pediatric OR Children) AND ("multiple electrolytes solution" OR MES OR balanced OR BS OR crystalloid OR buffered OR Plasmalyte OR "Ringer's Lactate" OR LR OR ringer funding OR Hartmann's OR sterio funding OR Kabilyte OR Normosol) AND ("normal saline" OR saline OR "0.9% saline" OR "0.9% sodium chloride" OR "0.9% NaCl") | 63 | No limitations applied |
