# Supplementary material for: Balanced Crystalloids Versus Normal Saline for Fluid Resuscitation in Pediatric Septic Shock: An Updated Systematic Review and Meta-Analysis
Source: J Clin Med Res. 2026 Oct 23;18(10):770–83. doi: 10.14740/jocmr6685 (PMC13719638; doi:10.14740/jocmr6685)
Supplement: Supplementary file 3 [file jocmr-18-10-770-s003.docx]

**Suppl 3.** Newcastle–Ottawa Scale assessment of the included observational cohort studies.

| **ID** | **Selection** | | | | **Comparability** | **Outcome** | | | **Quality Score** |
| --- | --- | --- | --- | --- | --- | --- | --- | --- | --- |
|  | **Representativeness of the exposed cohort** | **Selection of the non-exposed cohort** | **Ascertainment of exposure** | **Demonstration that the outcome of interest was not present at the start of the study** | **Comparability of cohorts on the basis of the design or analysis** | **Assessment of outcome** | **Was the follow-up long enough for outcomes to occur** | **Adequacy of follow-up of cohorts** |  |
| Sarmiento 2021 | * | * | * | * |  |  | * | * | Fair quality |
| Samransamruajki 2017 | * | * | * | * | ** | * |  | * | Good quality |
| Emrath 2017 | * | * | * | * | * | * | * | * | Good quality |
| Weiss 2017 | * | * | * | * | * | * | * | * | Good quality |
