# Supplementary material for: Balanced Crystalloids Versus Normal Saline for Fluid Resuscitation in Pediatric Septic Shock: An Updated Systematic Review and Meta-Analysis
Source: J Clin Med Res. 2026 Oct 23;18(10):770–83. doi: 10.14740/jocmr6685 (PMC13719638; doi:10.14740/jocmr6685)
Supplement: Supplementary file 16 [file jocmr-18-10-770-s016.docx]

**Suppl 16.** Summary of findings and quality of evidence based on the GRADE system

| **Certainty assessment** | | | | | | | **Summary of findings** | | | | | |
| --- | --- | --- | --- | --- | --- | --- | --- | --- | --- | --- | --- | --- |
| **No. of** **studies** | **Study design** | **Risk of bias** | **Inconsistency** | **Indirectness** | **Imprecision** | **Other considerations** | **No. of patients** | | **Effect** | | **Certainty** |  |
|  |  |  |  |  |  |  | **BS** | **NS** | **Relative** **(95% CI)** | **Absolute** **(95% CI)** |  |  |
| **All-cause mortality** | | | | | | | | | | | | |
| 9 | 5 RCTs + 4 cohorts | not serious | not serious | not serious | serious | TSA inconclusive; publication bias not assessed | 6,663 | 14,030 | RR 0.94 (0.83 to 1.06) | 7 fewer per 1,000 (19 fewer to 7 more) | ⊕⊕⊕◯ Moderate |  |
| 5 | 5 RCTs only | not serious | not serious | not serious | serious | TSA inconclusive; publication bias not assessed | 4,651 | 4,672 | RR 1.02 (0.86 to 1.20) | 1 more per 1,000 (6 fewer to 9 more) | ⊕⊕⊕◯ Moderate |  |
| **Acute kidney injury** | | | | | | | | | | | | |
| 8 | 5 RCTs + 3 cohorts | not serious | serious | not serious | serious | TSA inconclusive; publication bias not assessed | 6,648 | 14,010 | RR 0.84 (0.61 to 1.16) | 23 fewer per 1,000 (55 fewer to 23 more) | ⊕⊕◯◯ Low |  |
| 5 | 5 RCTs only | not serious | serious | not serious | serious | TSA inconclusive; publication bias not assessed | 4,651 | 4,672 | RR 0.94 (0.58 to 1.53) | 3 fewer per 1,000 (18 fewer to 23 more) | ⊕⊕◯◯ Low |  |
| **Major adverse kidney events within 30 days (MAKE30)** | | | | | | | | | | | | |
| 2 | 2 RCTs | not serious | not serious | not serious | serious | Estimate dominated by PRoMPT BOLUS; publication bias not assessed | 4,259 | 4,273 | RR 1.11 (0.87 to 1.41) | 3 more per 1,000 (4 fewer to 12 more) | ⊕⊕⊕◯ Moderate |  |
| **Renal replacement therapy** | | | | | | | | | | | | |
| 8 | 4 RCTs + 4 cohorts | not serious | not serious | not serious | serious | Benefit not robust in sensitivity analyses; publication bias not assessed | 6,652 | 14,019 | RR 0.74 (0.56 to 0.96) | 13 fewer per 1,000 (23 fewer to 2 fewer) | ⊕⊕⊕◯ Moderate |  |
| **Mechanical ventilation** | | | | | | | | | | | | |
| 3 | 2 RCTs + 1 cohort | not serious | not serious | not serious | serious | Publication bias not assessed | 390 | 403 | RR 0.97 (0.85 to 1.11) | 16 fewer per 1,000 (80 fewer to 58 more) | ⊕⊕⊕◯ Moderate |  |
| **Hyperchloremia** | | | | | | | | | | | | |
| 4 | 4 RCTs | not serious | not serious | not serious | not serious | Large, consistent biochemical effect; publication bias not assessed | 3,048 | 3,105 | RR 0.65 (0.61 to 0.69) | 174 fewer per 1,000 (194 fewer to 155 fewer) | ⊕⊕⊕⊕ High |  |
| **Hospital length of stay** | | | | | | | | | | | | |
| 4 | 3 RCTs + 1 cohort | not serious | serious | not serious | serious | Publication bias not assessed | 5,750 | 13,133 | MD 1.49 days (−1.65 to 4.62) | 1.49 days more (1.65 fewer to 4.62 more) | ⊕⊕◯◯ Low |  |
| **PICU length of stay** | | | | | | | | | | | | |
| 3 | 1 RCT + 2 cohorts | not serious | not serious | not serious | serious | Publication bias not assessed | 1,500 | 8,875 | MD 0.84 days (−0.27 to 1.95) | 0.84 days more (0.27 fewer to 1.95 more) | ⊕⊕⊕◯ Moderate |  |

GRADE approach. This table reproduces the pooled estimates shown in the current all-study figures. Risk of bias was judged not serious because the randomized trials were generally at low risk of bias and the observational cohorts were rated acceptable on the Newcastle–Ottawa Scale; therefore, no outcome was downgraded solely for risk of bias. Publication bias was not assessed when fewer than 10 studies contributed.

**Explanatory notes**

a) All-cause mortality (all studies): Nine studies (5 RCTs and 4 observational cohorts) were pooled. Risk of bias was not considered serious. Downgraded one level for imprecision because the 95% CI crossed the line of no effect and TSA remained inconclusive. Heterogeneity was absent (I²=0%).

b) All-cause mortality (RCTs only): Five RCTs were pooled (RR 1.02, 95% CI 0.86 to 1.20; I²=0%). Risk of bias, inconsistency, and indirectness were not serious. Downgraded one level for imprecision because the confidence interval crossed no effect and the RCT-only TSA remained inconclusive. Overall certainty: Moderate.

c) Acute kidney injury (all studies): Eight studies (5 RCTs and 3 observational cohorts) were pooled. Risk of bias was not considered serious. Downgraded for inconsistency because heterogeneity was substantial (I²=73.5%) and for imprecision because the 95% CI included clinically important benefit and harm; TSA remained inconclusive.

d) Acute kidney injury (RCTs only): Five RCTs were pooled (RR 0.94, 95% CI 0.58 to 1.53; I²=69.1%). Risk of bias and indirectness were not serious. Downgraded for inconsistency and imprecision because substantial heterogeneity persisted and the confidence interval included both benefit and harm; the RCT-only TSA remained inconclusive. Overall certainty: Low.

e) MAKE30: Two RCTs contributed. Downgraded for imprecision because the 95% CI crossed the line of no effect and the pooled estimate was dominated by the large PRoMPT BOLUS trial (I²=0%).

f) Renal replacement therapy: Eight studies (4 RCTs and 4 observational cohorts) were pooled. Risk of bias was not considered serious. Downgraded one level for imprecision/fragility because the apparent benefit was not robust in the additional sensitivity analyses requested by the reviewer, despite low heterogeneity.

g) Mechanical ventilation: Three studies (2 RCTs and 1 observational cohort) contributed. Risk of bias was not considered serious. Downgraded one level for imprecision because the 95% CI crossed the line of no effect (I²=0%).

h) Hyperchloremia: Four RCTs contributed. No downgrade was applied: the reduction was large and consistent, the 95% CI was narrow, and heterogeneity was absent (I²=0%).

i) Hospital length of stay: Four studies (3 RCTs and 1 observational cohort) contributed. Risk of bias was not considered serious. Downgraded for inconsistency (I²=65.2%) and imprecision because the 95% CI was wide and crossed no effect.

j) PICU length of stay: Three studies (1 RCT and 2 observational cohorts) contributed. Risk of bias was not considered serious. Downgraded one level for imprecision because the 95% CI crossed no effect; statistical heterogeneity was absent (I²=0%).

**Absolute effects:** For binary outcomes, absolute effects were calculated using the pooled control-group event risk from the studies contributing to the corresponding figure. BS, balanced crystalloids; NS, normal saline; RR, risk ratio; MD, mean difference; CI, confidence interval; RCT, randomized controlled trial; PICU, pediatric intensive care unit; TSA, trial sequential analysis.
