# Supplementary material for: Balanced Crystalloids Versus Normal Saline for Fluid Resuscitation in Pediatric Septic Shock: An Updated Systematic Review and Meta-Analysis
Source: J Clin Med Res. 2026 Oct 23;18(10):770–83. doi: 10.14740/jocmr6685 (PMC13719638; doi:10.14740/jocmr6685)
Supplement: Supplementary file 25 [file jocmr-18-10-770-s012.docx]

**Suppl 12.** Subgroup analysis of renal replacement therapy according to study design.
